# Asthma control, emergency visits, lung function and FENO in asthma and non-asthma in the West Sweden Asthma Study (WSAS)

**DOI:** 10.1186/2045-7022-3-S1-P20

**Published:** 2013-05-03

**Authors:** Jan Lötvall, Anders Bjerg, Linda Ekerljung, Bo Lundback

**Affiliations:** 1 Krefting Research Centre, University of Gothenburg, Sweden

## 

WSAS is a clinical epidemiological study, based on a random population of 30000 individuals living in West Sweden. After having answered a questionnaires, a random population was invited to extensive clinical phenotyping. We here report lung function FENO data from 954 asthmatics and 1030 non-asthmatics. Asthma was defined as “doctors diagnosis of asthma” and “ever asthma” + ”current asthma medication or symptoms common in asthma”. According to GINA criteria, 57.6% of asthmatics had controlled asthma, 29.3% partly controlled asthma and, 13.1% uncontrolled asthma. Weekly night-time asthma awakenings was reported by 12.6% of asthmatics. The prevalence of emergency visits over the last year due to asthma was 13.9% in non-smokers, 17.1% in ex-smokers, and 31.6 in current smokers (p=0.006 for trend). Mean %pred FEV1 was 105.3% in non-asthma and 96.9 in asthma. In the non-asthma group, 29.4% of individuals had FEV1 <100% predicted, whereas 52.6% of asthmatics had <100% predicted FEV1. In the non-asthma group, 4.6% of individuals had FEV1 <80% predicted, whereas 14.8% of asthmatics had <80% predicted FEV1. Mean FENO was 25.2 in asthma, and 19.9 in non-asthma (P<0.001). Low FENO (<20ppb) was observed in 62.8% of individuals in the non-asthma group, and 54% in the asthma group. High FENO (>50 ppb) was observed in 3% of the non-asthma group and 9.6% of the asthma group. Asthma with significant severity, leading to emergency visits, is common in the WSAS, and previous or current smoking increase that risk. Furthermore, close to every other individual with asthma report uncontrolled or partly controlled disease. Despite significant disease severity in this epidemiological setting, very few asthmatics express low lung function or very high exhaled FENO. Neither FEV1, nor FENO may be appropriate markers of disease severity in an epidemiological setting. However, WSAS may be utilized to further explore determinants of asthma severity and their risk factors, which will be reported at the meeting. This work was financed by the VBG Foundation against asthma/allergy.

